# A leadership-based framework for improving Saudi Arabian female participation in sports

**DOI:** 10.3389/fspor.2023.1283842

**Published:** 2023-11-03

**Authors:** Mamdouh Dawish Alruwaili

**Affiliations:** Department of Sports Science & Physical Education, College of Arts, Jouf University, Sakakah, Saudi Arabia

**Keywords:** Saudi Arabia, participation, engagement, females, sports, barriers, leadership styles, transformational leadership

## Abstract

**Study purpose:**

To analyze the relationship between the leadership styles and sports engagement among female sport persons in Saudi Arabia and proposes a framework model for improving Saudi Arabian female participation in sports.

**Methods:**

This study adopted an online cross-sectional survey design for achieving the research aim. Survey instruments included multi-factor leadership questionnaire (MLQ) and Utrecht Work Engagement Scale (UWES). The study participants included adult female sports persons from various sports clubs in Saudi Arabia. A total of 329 responses were received, out of which 35 were incomplete; therefore, a total of 294 responses were considered for the data analysis.

**Results:**

Transformational leadership style was identified to be the most preferred style compared to transactional and laissez-fare leadership styles. Older participants (>25 years) perceived transformational leadership scales including inspirational motivation, intellectual stimulation, and individual consideration to be more effective (*p* < 0.05) compared to younger participants. Transformational leadership has strong positive correlation with all engagement scales (as correlation coefficient “r” was greater than 0.7, *p* < 0.01).

**Conclusion:**

Inspirational motivation could be an effective approach in increasing the female participation in Saudi Arabia, as they are mentally unprepared due to the experience of socio-cultural restrictions for decades.

## Introduction

As part of the Vision 2030 program, Saudi Arabia is transitioning away from an economy that is dependent on oil and toward one that is focused on information (1). During this procedure, numerous issues were taken into consideration, one of which was the promotion of women's empowerment, particularly through the encouragement of female participation in sports. Participation in sports enhances physical activity among the individuals. The concept of “physical activity” encompasses all forms of body motions generated by skeletal muscles that necessitate energy expenditure surpassing the baseline level (2). There is a growing body of literature that suggests a strong correlation between frequent participation in physical activity and its positive impact on an individual's overall health and well-being (3, 4). Moreover, it is worth noting that several governmental investigations and consensus statements have underscored the fundamental significance of physical activity in both individual and communal contexts (5–7). Physical inactivity and sedentary behavior have been recognized as significant contributors to various chronic non-communicable diseases, including diabetes mellitus, obesity, cardiovascular disorders, some types of cancer, osteoporosis, and mental impairments (8, 9). Moreover, it has been established that insufficient engagement in physical exercise is associated with untimely death (10). As a result, the monitoring of physical activity or sports engagement among the individuals has progressively emerged as a matter of public health significance. It has been observed that the focus on women environment was mainly on their involvement, improvement, and achieving their rights, which needs a change in socio-cultural, political, legal, economic, and environmental systems (11). However, there are a variety of barriers that prevent Saudi women from participating in sports. These obstacles include socio-cultural, family, and institutional aspects (12, 13).

One of the most significant characteristics of cultures that has been the topic of debate for millennia everywhere in the world is the way in which women are treated unfairly in those communities. Even though gender inequality has been somewhat addressed in many countries because of policy and regulations, prejudice remains deeply ingrained in the society and culture of a select number of nations. It is a factor of societal and cultural norms reflecting the subservient roles of women in the society (14). For example, in a few countries such as Pakistan, Afghanistan, and Saudi Arabia, the practice of patriarchal conjugal relationships reflects the incorrect perception of linking women's discrimination to religion. In these countries, the practice of patriarchal conjugal relationships reflects the ill perception of linking women discrimination to religion. According to OECD (15), Saudi Arabia is one of the nations in which several problems have been highlighted about women. These problems include discrimination in the family, threats to physical integrity, a lack of productive and financial resources, restrictions on civil freedoms, and more. In addition, discrimination in fundamental rights such as work, healthcare, and personal development were just a few parts of the concerns that were harming the empowerment of women in Saudi Arabia (16, 17). In most cases, the influence of these variables prevents women from accessing a variety of possibilities that can help them achieve their goals and slows down the process of their growth. One of these areas in the country that women are not allowed to participate in, which has been the case for quite some time now, is sports.

According to a recent study conducted by Al-Dubayan (17), inequalities in organizations are still prevalent in Saudi Arabia. These inequalities are particularly prevalent in the adoption of customs such as wasta and asabiyyah, in which advantages were given to family members or those belonging to the same status. In Saudi Arabia, sports organizations are the same as any other kind of organization, just like any other kind of organization, they involve a variety of tasks such as training, management of sports activities, and so on. On the other hand, as part of its Vision 2030 program, the Saudi government has undertaken several measures at the national level to improve the quality of life and health of women and girls, and at the international level, it has increased the visibility of Saudi female athletes (18). Because of this, the number of women who manage sports clubs and teams as well as the number of women who participate in sports has increased by 56%, which is a promising indicator for the expansion of options available to women who are interested in participating in sports (19). On the other hand, there are still obstacles that make it difficult for women to participate in sports. To allow women to participate in a variety of sports, for instance, it was necessary to consider numerous socio-cultural elements, such as gender segregation and Islam (20, 21). In addition, the attitudes of family members, the location of the home (whether it be urban or rural), and the degree of education were shown to be the primary obstacles for female engagement in sports (21). In a similar vein, in educational settings, physical fitness, social experience, and formal competition all have a beneficial effect on physiological experience, which in turn has a positive effect on the attitude of female students towards sports (22). Therefore, there may be a variety of areas in which the obstacles that prevent women from participating in sports can be recognized based on the research that is done, and these areas need to have competent leadership handled.

Although, there is issues with gender equality in sports participation globally, its impact was mainly observed in the Middle East till the past decade (23). Furthermore, it was observed that when compared to other Arab or Muslim women, Saudi women are subject to a greater number of limitations since they are prohibited (legally or culturally) from doing many things by themselves and are required to have a male guardian present to participate in some activities (24). Young Arabic Muslim women who participate in sports are perceived as taking on a challenge to the limits of their ethnic identities when compared to their peers who do not (24). Young Arabic Muslim women who firmly position themselves within the context of their ethnic identities are not interested in sports since participating in athletics is not viewed as respectable femininity among Arabic Muslim culture (25). Even though the Kingdom of Saudi Arabia places a strong emphasis on the importance of protecting the female population, the lack of physical education for girls in schools, which only stopped very recently, the limited number of fitness centers and gyms for women, a particular body image, and the prevalence of women staying at home and leading rather passive or static lives have all contributed to a somewhat unhealthy lifestyle among women (26). In the past, Saudi women have not had access to physical education or sports, which is evidenced by the poor health status they currently suffer from (44% obesity rate), which is significantly higher than the national average (35.4%) and nearly three times as high as the worldwide average (13%) (13). Although, significant changes have been introduced in the past few years in kingdom for promoting female physical activity and health, there is a change being observed in women participation in sports and physical activity, but they still face issues in different contexts (27, 28). A recent study (29) suggested an ambitious policy that was well-founded, as well as the achievement of several goals in Saudi Arabia. It was discovered that the most obvious results have thus far been the rapid, albeit modest cultural change in terms of physical activity, allowing women to participate in sports and raising the level of physical activity among Saudis. This transformation was one of the most evident achievements. Despite this, there are still some objectives that have not been met, most notably those associated with the participation of women in sports. In conclusion, there has been some movement toward the actualization of the policy regarding the promotion of physical activity; however, there are several factors that exist that have hampered achievement in particular domains, revealing a lack of effective leadership practices in motivating female participation in sports (29).

With the change being observed, it is essential that there is a need to increase research in this area to better analyze the policies, and the impact of various factors on women participation in sports in Saudi Arabia. Such research studies can have various benefits, as it can aid in promoting gender equality, promoting women's empowerment, improving health outcomes, and reaping economic and social benefits. It enables policymakers, sports organizations, and leaders to identify strategies that encourage female participation and create a more inclusive and equitable sports landscape. However, the number of studies focusing on the women and sports participation is very low as observed in a recent systematic review (30). Therefore, to address such gaps, this study aims to analyze the relationship between the leadership styles and sports engagement among female sport persons in Saudi Arabia and proposes a framework model for improving Saudi Arabian female participation in sports.

## Hypothesis development

The relationship between transformational leadership style (a leadership style that focuses on inspiring and motivating followers to achieve their full potential and exceed their own expectations) and engagement was studied in various contexts such as manager and employee relations. Studies (31–33) have identified that transformational leadership contributed to various aspects such as needs satisfaction, less job strain, increased autonomy, and support, leading to a positive correlation between transformational leadership and employee engagement. In the context of sports, transformational leadership was related with athletes well-being and satisfaction (34), leading to a positive correlation with sports/physical activity engagement (35, 36). In this context, this study considers following hypothesis.

*H1: Transformational leadership exhibits strong positive correlation with engagement in sports.*
  Similarly, studies (37–42) have identified that transactional leadership (a leadership style that emphasizes the exchange of rewards and punishments between the leader and their followers based on the completion of tasks and meeting performance expectations) reflect moderate positive correlations with engagement in physical activity as it promotes non-autonomous motivation; but laissez-faire leadership (characterized by leaders who give their team members a high degree of autonomy and decision-making authority) reflected negative correlations with engagement in physical activities. In this context, the following hypotheses are formulated.
*H2: Transactional leadership exhibits moderate positive correlation with engagement in sports.*
*H3: Laissez-faire leadership exhibits negative correlation with engagement in sports.*

## Methods

This study adopted an online cross-sectional survey design for achieving the research aim.

### Questionnaire design

The first part of the questionnaire focuses on collecting the demographic information of the participants, which includes age, education, and sports experience. The second and third parts of questionnaires includes two pre-validated and academically recognized survey questionnaires respectively, which include: multi-factor leadership questionnaire (MLQ) (43) and Utrecht Work Engagement Scale (UWES) (44). MLQ is one of the prominent questionnaires that is used for identifying leadership styles in different settings (45); and UWES, although developed for assessing work engagement in organization, it is adopted in sports environment for assessing sports engagement (44). Using these questionnaires, individual leadership scales and sports engagement scales can be correlated to test the hypothesis. The MLQ consists of 21 questions, each of which must be scored on a Likert scale ranging from 0 (never) to 4 (very often), with 1 denoting never and 5 denoting never more than once in a while. The focus of items 1–12 is on several forms of transformational leadership, which can be further subdivided into idealized influence (items 1–3), inspiring motivation (items 4–6), intellectual simulation (items 7–9), and individual attention (items 10–12). Items 13–18 are associated with transactional leadership styles, which can be subdivided into management by exception (items 16–18) and contingent reward (items 13–15). Items 19–21 are associated with a leadership style known as laissez-faire. UWES-9 has 15 items which are grouped under vigor, dedication, and absorption categories. The participants' degrees of agreement or disagreement with each question on the UWES-9 are reflected by their placement on a scale with five points, ranging from 1 (Never) to 5 (Always). The UWES-9 consists of 15 items, five of which are categorized as belonging to the vigor category, five to the dedication category, and five to the absorption category, respectively.

Two Arabic translators were employed to translate the questionnaire from English to Arabic. The individual translated versions are compared and a final version is developed by the authors. The final version is reviewed by the academic professors from Language and cultural studies department and minor changes related to grammar were suggested, which were implemented. After having the questionnaire translated into Arabic, a pilot study was carried out with 21 participants. The results, which showed a Cronbach's alpha of better than 0.70 for all items, suggested good internal reliability and consistency (38). Using Google Survey, an online version of the survey questionnaire was created, and a link to that online version is being established for the purpose of data collection.

### Participants, recruitment, and sampling

As the study is focused on female sportspersons, the participants in this study included female sportspersons in Saudi Arabia aged more than 18 years. The participants from the study were recruited from the five sports clubs in Saudi Arabia. Five sports clubs were contacted for participation in this study, to whom an invitation email with survey link was sent. The email was then forwarded to the members of respective female sports clubs. In addition, snow-ball sampling (45) was used, where a request was placed in the email to forward the link to their colleagues who are female sports persons.

Considering the 14 million Saudi female population (46), estimated sample was calculated using Cochran's formula (47), at 95% CI and 5% of Margin of error, giving an estimated sample of 384 participants.

### Data collection and analysis

The data was collected online using an online-administered survey questionnaire. The invitation email was sent to 594 female sports persons, from five sports clubs. A total of 329 responses were received, out of which 35 were incomplete; therefore, a total of 294 responses were considered for the data analysis.

The data was analyzed with SPSS version 20.0, and a variety of statistical methods, such as *t*-tests and Pearson's correlation, were utilized. To remove any potential sources of bias from the analysis of the results, missing data were eliminated. The key finding of the investigation is a correlation between leadership style and the level of participation displayed by sportspeople.

## Results

As the study is focused on analyzing female sports persons perceptions of leadership styles and sports engagement, only 294 female sports persons are included (See Table 1). Considering the education attributes of the participants, majority of them were diploma holders (44.9%), followed by bachelor's degree holders (40.1%), master's degree holders (10.2%, and 4.8% participants with primary/secondary school education. Considering the age factor, most of the participants were distributed across three age groups including 22–25 years (31.3%), 18–21 years (29.3%), and 26–29 years (22.8%). About 9.5% were aged between 30 and 33 years, and 7.1% were aged more than 33 years. In relation to experience in sports, 43.2% participants had three to five years of experience, followed by 40.5% having six to ten years of experience, and 16.3% having more than ten years of experience.

**Table 1:** Participants demographics.

Variable	Demographic characteristics	Frequency counts	Percentage
Education	Primary/Secondary education	14	4.8%
	Diploma	132	44.9%
	Bachelor's degree	118	40.1%
	Master's degree	30	10.2%
Age	18–21	86	29.3%
	22–25	92	31.3%
	26–29	67	22.8%
	30–33	28	9.5%
	>33	21	7.1%
Experience in sports (participating in sports related activities for how many years?)	3–5 years	127	43.2%
	6–10 years	119	40.5%
	>10 years	48	16.3%

Considering the perceptions on different leadership styles, transformational leadership was most preferred by the participants, as it can be observed from mean rating or average scores for sub-scales including idealized influence (3.11 out of 5), inspirational motivation (3.16), intellectual stimulation (3.04), individual consideration (3.11). Transactional leadership styles are less preferred as the mean ratings of sub-scales reflected low to medium preferences: contingent rewards (2.66) and management by exception (2.65). Laissez-faire leadership was least preferred by the participants as it achieved lower ratings (2.47) compared to other leadership styles. All engagement sub-scales vigor (2.71), dedication (2.72), and absorption (2.72) indicated low to medium levels of engagement in sports activities.

To further analyze the results by participants groups (see Table 2), *t*-tests were conducted. Statistically significant differences (*p* < 0.05) were observed between younger and older participants in relation to transformational leadership scales including inspirational motivation (*p* = 0.03, *p*, 0.05), intellectual stimulation (*p* = 0.02, *p* < 0.05), and individual consideration (*p* = 0.03, *p* < 0.05). Older participants preferred transactional leadership styles more than the younger participants. However, no statistically significant differences were observed among the participants with different levels of sports experience in relation to transformational and laissez-faire leadership styles. Statistically significant difference (*p* < 0.05) was observed with respect to transactional leadership sub-scale: management by exception (*p* = 0.03, *p* < 0.05) among the participants with different experience levels. Participants with experience over five years preferred management by exception factor more than the participants with lower experience levels.

**Table 2:** Perceptions about leadership styles grouped by age and sports experience variables.

			*N*	Mean	SD	*t*-value	*p*-value
Idealized influence	Age	≤25 years	178	3.02	1.0	1.6265	.0525
		>25 years	116	3.23	1.18		
	Experience	3–5 years	127	3.04	1.34	0.8271	0.2044
		> 5 years	167	3.15	.97		
Inspirational motivation	Age	≤25 years	178	3.08	.8	1.8795	0.0306*
		>25 years	116	3.29	.87		
	Experience	3–5 years	127	3.12	.95	0.7618	0.2234
		>5 years	167	3.2	.74		
Intellectual stimulation	Age	≤25 years	178	2.91	1.03	2.8574	0.0023*
		>25 years	116	3.25	1.01		
	Experience	3–5 years	127	3.07	1.13	0.3468	0.3645
		>5 years	167	3.03	.99		
Individual consideration	Age	≤25 years	178	3.02	.97	1.8088	0.0358*
		>25 years	116	3.23	1.05		
	Experience	3–5 years	127	3.13	1	0.4059	0.3421
		>5 years	167	3.08	1.02		
Contingent reward	Age	≤25 years	178	2.62	1.16	.7924	0.2944
		>25 years	116	2.72	1.18		
	Experience	3–5 years	127	2.59	1.21	1.0629	0.1443
		>5 years	167	2.73	1.14		
Management by exception	Age	≤25 years	178	2.59	1.12	1.2532	0.1056
		>25 years	116	2.75	1.03		
	Experience	3–5 years	127	2.52	1.13	1.881	0.0305*
		>5 years	167	2.76	1.04		
Laissez-faire	Age	≤25 years	178	2.45	1.21	0.4424	0.3292
		>25 years	116	2.51	1.17		
	Experience	3–5 years	127	2.44	1.22	0.5635	0.2867
		>5 years	167	2.51	1.16		

* Statistically significant difference.

Interestingly, no statistically significant differences were observed between the participants groups with respect to sports engagement (see Table 3).

**Table 3:** Perceptions about sports engagement grouped by age and sports experience variables.

Engagement factor	Variable	Group	*N*	Mean	SD	*t*-value	*p*-value
Vigor	Age	≤25 years	178	2.68	1.32	0.6697	0.2518
		>25 years	116	2.78	1.16		
	Experience	3–5 years	127	2.74	1.41	0.4103	0.3409
		>5 years	167	2.68	1.15		
Dedication	Age	≤25 years	178	2.64	1.24	1.4977	0.0677
		>25 years	116	2.84	1.31		
	Experience	3–5 years	127	2.71	1.31	0.0924	0.4632
		>5 years	167	2.73	1.25		
Absorption	Age	≤25 years	178	2.63	1.21	1.6223	0.0531
		>25 years	116	2.85	1.28		
	Experience	3–5 years	127	2.7	1.26	0.2541	0.3997
		>5 years	167	2.74	1.22		

Table 4 presents correlations between the leadership styles and sports engagement sub-scales. It is evident that transformational leadership has strong positive correlation with all engagement scales (as correlation coefficient “r” was greater than 0.7, *p* < 0.01). Furthermore, correlations between transactional leadership and laissez-faire leadership styles were identified to be having moderate relationship (as correlations ranged between 0.5 and 0.6) with all sub-scales of engagement levels including vigor, dedication, and absorption.

**Table 4:** Correlations between MLQ and UWES sub-scales.

	Vigor	Dedication	Absorption
Idealized influence	.736**	.744**	.731**
Inspirational motivation	.764**	.798**	.793**
Intellectual stimulation	.734**	.768**	.772**
Individual consideration	.727**	.764**	.769**
Contingent reward	.557**	.544**	.535**
Management by exception	.553**	.547**	.541**
Laissez-faire	.518**	.507**	.511**

** Correlation is significant at the 0.01 level (2-tailed).

## Discussion

The findings in this study indicated that sports managers exhibited transformational leadership styles far better than transactional and laissez-faire leadership styles. Furthermore, transactional leadership styles and laissez-faire leadership styles were also adopted, but to a lower extent. Although, all the three leadership styles were exhibited, sports managers were more inclined towards transformational leadership styles. It may be possible that managers exhibit different leadership styles according to the working conditions (48–51), because of which different styles can be observed by the sports persons. However, previous studies (52, 53) observed that Saudi women unknowingly act in accordance with male ideals, as they are culturally nourished with the thinking of male superiority over females; and as such, they tend to prefer masculine leadership, which is characterized by assertiveness and competitiveness. However, the sports managers for female sports persons include female managers (54, 55), who may adopt transformational leadership styles which are characterized by support, motivation, and cooperation rather than masculine leadership styles, which is evident from the high mean scores for transformational leadership sub-scales in this study.

Among the transformational leadership sub-scales, inspirational motivation was observed to be the most frequently displayed sub-scale. Given the various socio-cultural barriers for females in Saudi Arabia (23–28) from entering sports, motivational approach could be an effective strategy to boost the morale and engagement of the female sports persons in Saudi Arabia. Accordingly, inspirational motivation exhibited strong correlation with sports engagement sub-scales including vigor, dedication, and absorption; compared to other transactional leadership sub-scales. Intrinsic motivation is one of the benefits of adopting transformational leadership. Studies (56–59) have observed that motivation related intervention such as gamification can enhance positive attitudes and emotions of the individuals to engage in physical activities. These findings suggest that transformational leadership style could be more favorable in increasing sports engagement compared to other styles as it fosters motivation among the sportspersons through enhanced support from the leaders. In addition, skills and sports literacy/awareness were also identified to be significant factors influencing the sports engagement (60, 61), which require transformative approach which focuses on support, change management, continuous improvement, and skills development through an inspirational motivation. Supporting these outcomes, the hypotheses H1 was proved to be true. Similarly, hypotheses H2, indicating moderate positive correlation of transactional leadership on engagement in sports was observed to be true supporting the findings from (37–40). However, in contrast to the findings in (37–42), laissez-faire leadership exhibited moderate positive correlation with engagement in sports, resulting in hypotheses H3 to be determined as false. These contrasting results may be due to the prevalence of laissez-faire and paternalistic leadership styles in Saudi Arabia since early childhood in different contexts (62, 63).

However, several barriers including lack of time, safety, parental support, policies, access to sport and PA facilities, and transportation, as well as climate were observed for participation in physical activities among females in Saudi Arabia (64). Furthermore, the weekly physical activity levels of Saudi citizens aged above 15 years was identified to be 13% in 2015, 19% in 2019, which is very low compared to the target of 40% set by the country, indicating poor physical activity levels among citizens (65). Therefore, there is a need for strong support, effective and efficient policies from the government to foster the change in attitudes of the people in society towards the participation of females in physical activities and sports. The initiatives led by Vision 2030 has contributed to the increase in female participation in physical activities and sports in Saudi Arabi. For instance, establishment of female sports clubs, easing regulations for female participation in sports, deploying an operational process for sustainable and inclusive sports ecosystems launched by HRH Princess Reema bint Bandar Al Saud, have greatly contributed to the female participation in sports in the last few years (66). The changes being introduced recently could be the factor affecting the differences in the younger groups perceptions about transformational leadership styles as observed from the findings. Studies (30, 67) have also highlighted the importance and the need for workshops, training and support, motivation in educational institutions such as schools and universities to foster the change in attitudes of the people ad increase the participation in sports and physical activities. As a result, younger students may increase their participation, and the need for motivation may be slightly replaced with the need for stimulation, influence, and consideration.

In Saudi Arabia, only 17.4% of the population are practicing sports or physical activity for more than 150 min a week (67), which is less compared to 32% in European Union (68) and 22% in the USA (69). Few individuals participate in sports such as football (20.5%), swimming (5.3%), running (3.7%), and Swedish exercises (a system of active and passive exercises that use different muscles and joints of the body) (8.5%) in Saudi Arabia (70), indicating low engagement levels. Therefore, there is an urgent need to improve sports engagement in the Kingdom.

**Figure 1:**
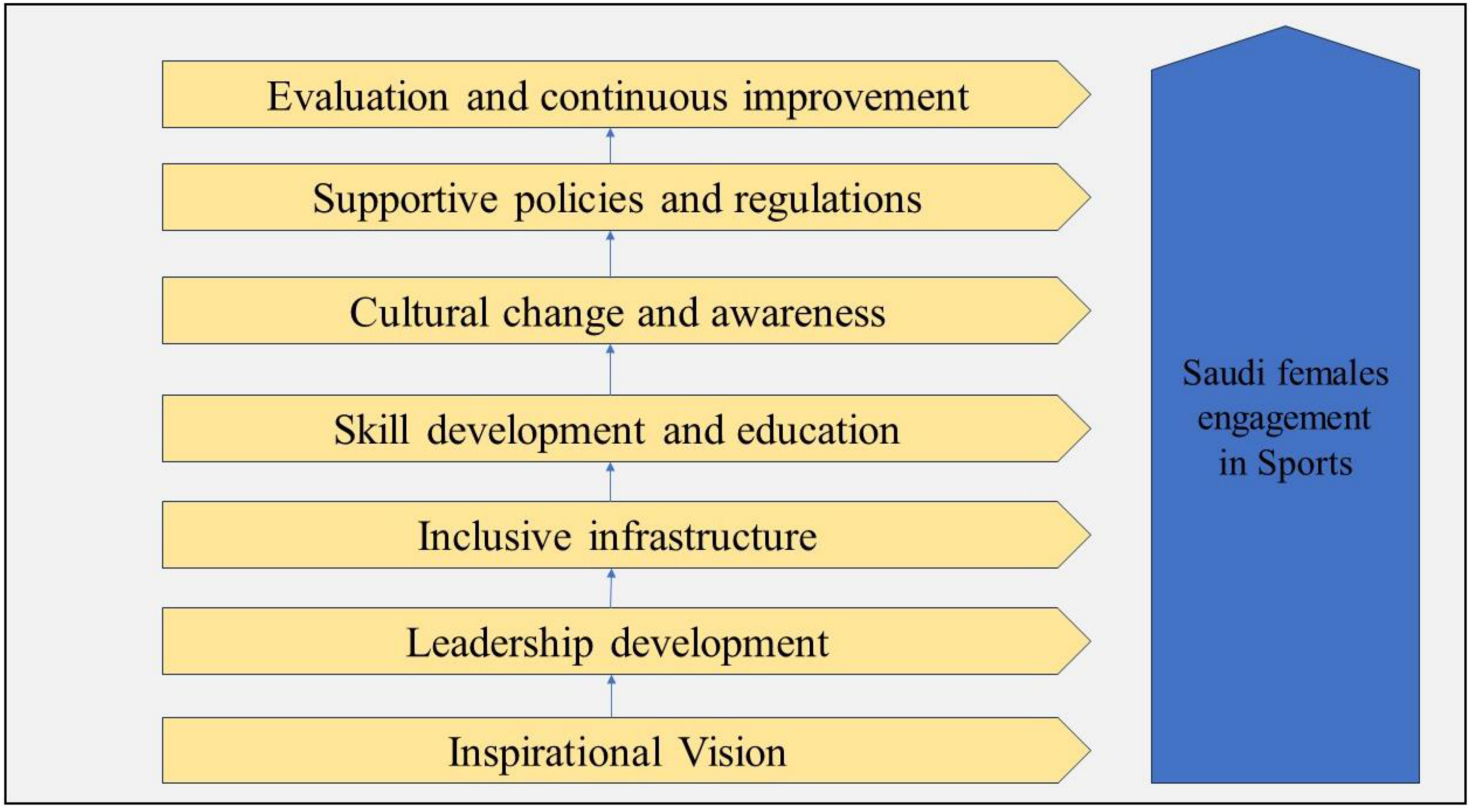
Framework model for promoting participation and engagement of Saudi females in sports.

Based on the discussion, a framework model is presented in Figure 1 to promote the participation and engagement of females in sports in Saudi Arabia.

The framework has seven main components, which are explained below:

1. Inspirational Vision:
  The first step in the transformational leadership model is to establish an inspiring vision that emphasizes the value and benefits of female participation in sports. This vision should be communicated effectively to various stakeholders, including government officials, sports organizations, community leaders, and families. By illustrating the positive impact of women's involvement in sports on individual well-being, health, personal growth, and national development, the model aims to generate enthusiasm and support.
2. Leadership Development:
  To drive the transformation, it is crucial to develop a strong cohort of transformational leaders who are passionate about promoting female participation in sports. These leaders should be selected from diverse backgrounds, including sports, academia, government, and community organizations. Specialized leadership development programs can be designed to enhance their knowledge, skills, and understanding of gender issues in sports. The training should focus on empowering leaders to challenge societal norms, advocate for change, and create opportunities for female athletes.
3. Inclusive Infrastructure:
  Creating a supportive and inclusive infrastructure is essential for increasing female participation in sports. This includes building sports facilities that cater to the specific needs of women, such as women-only gyms, swimming pools, and sports clubs. Additionally, promoting accessible and safe transportation options for female athletes will help overcome logistical barriers. Collaboration with sports organizations and private sector entities can be encouraged to invest in infrastructure development, ensuring it aligns with the specific requirements of female athletes.
4. Skill Development and Education:
  To encourage women to engage in sports, it is vital to provide comprehensive skill development and educational opportunities. Establishing sports academies and training programs exclusively for women, facilitated by experienced coaches and mentors, will foster talent, and empower aspiring athletes. Furthermore, incorporating physical education programs in schools and universities that prioritize gender equality in sports can help shape positive attitudes towards female participation from a young age.
5. Cultural Change and Awareness:
  Addressing deep-rooted cultural barriers is crucial for long-term success. The transformational leadership model aims to initiate a cultural shift by promoting awareness campaigns and engaging influential figures, such as religious leaders, celebrities, and media personalities. These campaigns should highlight the accomplishments and success stories of female athletes, challenge gender stereotypes, and emphasize the importance of equal opportunities in sports. Collaborations with media outlets can be established to ensure widespread coverage of women's sports events and achievements.
6. Supportive Policies and Regulations:
  To ensure sustainability, supportive policies and regulations need to be implemented. Government entities and sports organizations should work together to formulate policies that eliminate gender-based discrimination, provide equal funding and resources for female sports programs, and enforce strict measures against gender-based harassment and violence in sports settings. These policies should also mandate gender diversity on decision-making bodies to ensure female voices are heard.
7. Evaluation and Continuous Improvement:
  Regular evaluation and feedback mechanisms are essential to assess the effectiveness of the transformational leadership model. By collecting data on female participation rates, athlete satisfaction, infrastructure utilization, and public perception, areas of improvement can be identified and adjustments made accordingly. This ongoing assessment will ensure the model remains adaptable and responsive to evolving needs and challenges.

### Implications and limitations

There are few limitations which can be identified in this study. Firstly, the study population only focuses on the female sports persons, and does not include female students from school or universities, as a result, the findings in this study should be generalized with care. Secondly, it is important that the results of this study be viewed and generalized with care as there may be inevitable and unaccountable self-selection bias in the data.

There are various implications of the findings derived from this study. The findings in this study can be used by the decision-makers in developing strategies for attracting the female population in engaging physical activity by deploying leaders with transformational leadership, especially those who can motivate effectively. Secondly, these findings can be used to improve the quality of life of Saudi residents by increasing sports engagement, as studies have observed various health benefits such as improved physical and mental health (71, 72) and reduced risk of chronic illnesses (73, 74). Thirdly, this study contributes to the lack of female sports research study in Saudi Arabia by providing significance of the relationship between leadership styles and sports engagement among females in Saudi Arabia.

## Conclusion

Understanding the relationship between leadership styles and sports engagement among females in Saudi Arabia is important because of the achievement of Vision 2030 goals to increase female participation in all activities and to achieve sustainable sports system. However, the existence of various barriers for female participation highlighted the need for adopting inspirational motivation approach reflecting the transformational leadership practice by the sports managers. Thus, the findings indicated that transformational leadership is an effective approach to increase female participation and engagement in sports in Saudi Arabia.

## Data Availability

The raw data supporting the conclusions of this article will be made available by the author, without undue reservation.
